# Baseline assessment of the WHO/UNICEF/UNFPA maternal and newborn quality-of-care standards around childbirth: Results from an intermediate hospital, northeast Namibia

**DOI:** 10.3389/fped.2022.972815

**Published:** 2023-01-09

**Authors:** Gloria Mutimbwa Siseho, Thubelihle Mathole, Debra Jackson

**Affiliations:** ^1^ Faculty of Community and Health Sciences, School of Public Health, University of the Western Cape, Bellville, South Africa; ^2^ Maternal Newborn and Child Health, United Nations Children s Fund (UNICEF), Windhoek, Namibia; ^3^ Epidemiology and Public Health, London School of Hygiene and Tropical Medicine, London, United Kingdom

**Keywords:** WHO/UNICEF/UNFPA, quality-of-care, maternal and newborn, childbirth, Namibia

## Abstract

**Background:**

Quality of care around childbirth can reduce above half of the stillbirths and newborn deaths. Northeast Namibia’s neonatal mortality is higher than the national level. Yet, no review exists on the quality of care provided around childbirth. This paper reports on baseline assessment for implementing WHO/UNICEF/UNFPA quality measures around childbirth.

**Methods:**

A mixed-methods research design was used to assess quality of care around childbirth. To obtain good saturation and adequate women opinions, we purposively sampled the only high-volume hospital in northeast Namibia; observed 53 women at admission, of which 19 progressed to deliver on the same day/hours of data collection; and interviewed 20 staff and 100 women who were discharged after delivery. The sampled hospital accounted for half of all deliveries in that region and had a high (27/1,000) neonatal mortality rate above the national (20/1,000) level. We systematically sampled every 22nd delivery until the 259 mother–baby pair was reached. Data were collected using the Every Mother Every Newborn assessment tool, entered, and analyzed using SPSS V.27. Descriptive statistics was used, and results were summarized into tables and graphs.

**Results:**

We reviewed 259 mother–baby pair records. Blood pressure, pulse, and temperature measurements were done in 98% of observed women and 90% of interviewed women at discharge. Above 80% of human and essential physical resources were adequately available. Gaps were identified within the WHO/UNICEF/UNFPA quality standard 1, a quality statement on routine postpartum and postnatal newborn care (1.1c), and also within standards 4, 5, and 6 on provider–client interactions (4.1), information sharing (5.3), and companionship (6.1). Only 45% of staff received in-service training/refresher on postnatal care and breastfeeding. Most mothers were not informed about breastfeeding (52%), postpartum care and hygiene (59%), and family planning (72%). On average, 49% of newborn postnatal care interventions (1.1c) were practiced. Few mothers (0–12%) could mention any newborn danger signs.

**Conclusion:**

This is the first study in Namibia to assess WHO/UNICEF/UNFPA quality-of-care measures around childbirth. Measurement of provider–client interactions and information sharing revealed significant deficiencies in this aspect of care that negatively affected the client’s experience of care. To achieve reductions in neonatal death, improved training in communication skills to educate clients is likely to have a major positive and relatively low-cost impact.

## Introduction

Globally, during 2020, almost 47% of under-five deaths were among neonates (1), with neonatal deaths reported to be declined at a slower pace. The increase in facility deliveries in lower- and middle-income countries (LMICs) will not result in reduced maternal and newborn deaths (1–3) unless quality healthcare is improved. This includes the postnatal period (3–5). Improving quality-of-care standards during childbirth can reduce 61% of newborn deaths; however, half of the intrapartum stillbirths and maternal deaths result from poor quality care (3, 5, 6). Also, providing quality care is beneficial beyond survival as it prevents antepartum and intrapartum complications, supporting quality life for mothers and newborns (7). The time around birth and the first 24 h after birth (8, 9) remain the most vulnerable periods for mothers and newborns. The first month of life is the most susceptible period for child death (1, 10). This is despite the increasing evidence that healthcare quality plays a crucial role in promoting human rights, determining and improving health outcomes (3, 8, 11–14).

The WHO/UNICEF/UNFPA developed eight maternal and newborn quality standards in response to the growing need to improve the quality of maternal and newborn care during childbirth (Supplementary Table S1 and Figure S1). The standards address quality-of-care domains on the provision of care and experience of care. The provision of care includes (1) evidence-based practice for routine care and management of complications; (2) actionable information systems; and (3) functioning referral systems, while the experience of care includes (4) effective communication; (5) respect and preservation of dignity; and (6) emotional support. The last two standards are cross-cutting domains and include (7) competent, motivated personnel; and (8) availability of essential physical resources.

**Figure 1:**
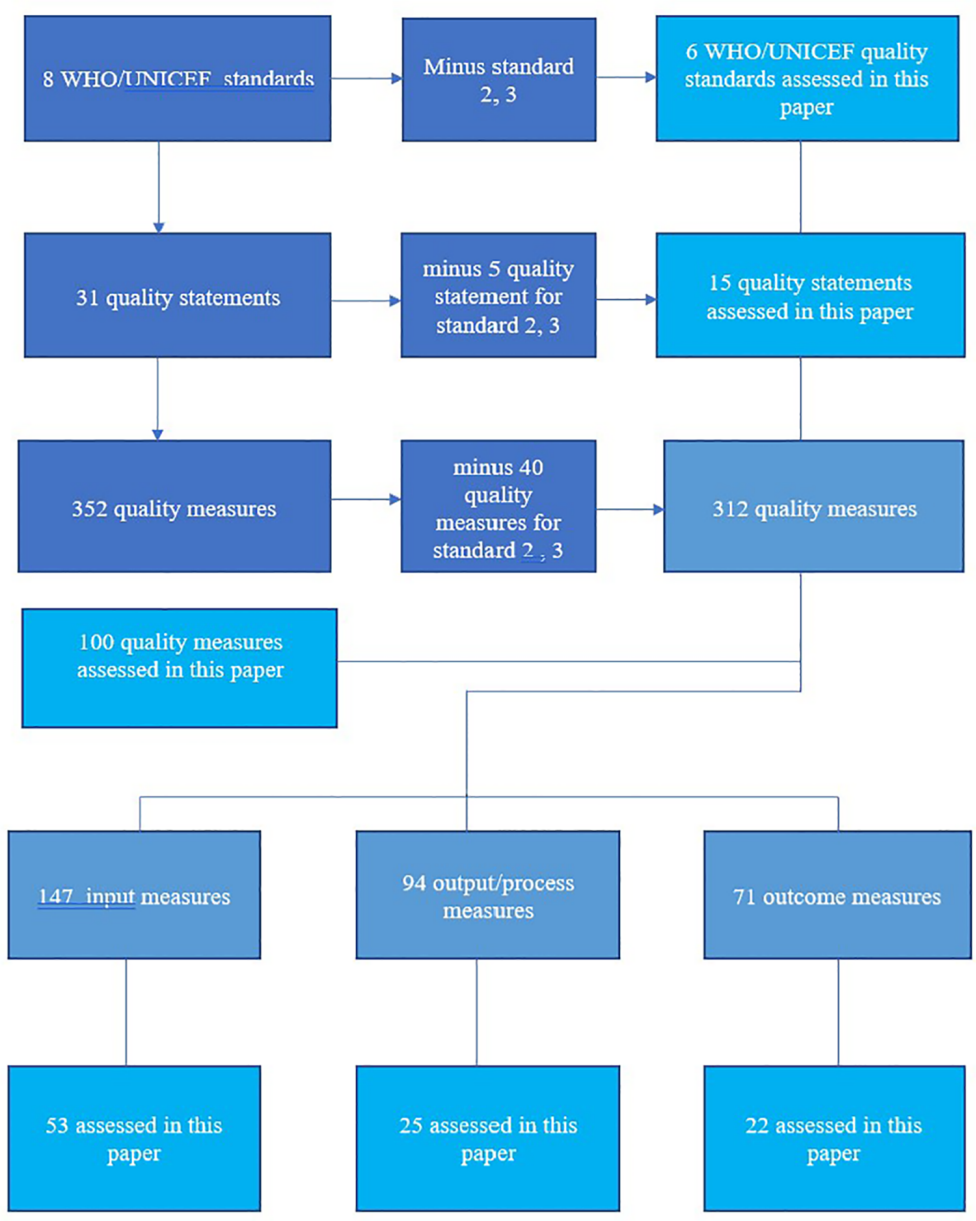
Standards, statements, and measures under review in this paper.

In Namibia, a baseline assessment of implemented WHO/UNICEF/UNFPA quality-of-care measures (5) for reducing preventable newborn deaths is not available. Thus, this study reports preliminary findings of the quality-of-care interventions implemented around childbirth. We assessed the quality-of-care standards around childbirth and gauged the results against the WHO/UNICEF/UNFPA quality standards, statements, and measures using the Every Mother Every Newborn assessment tool (15). Benchmarking is crucial in identifying quality gaps and opportunities to inform strategic lifesaving interventions around childbirth. Of crucial note is that in Namibia, the WHO/UNICEF/UNFPA standards are not standalone. They provide innformation about the development of the 2021 national quality management strategic plan and policy. This means that by the time of data collection, national draft strategies and guidelines had limited alignment with the standards. This study therefore aims to describe the preliminary results for implementing the WHO/UNICEF/UNFPA standards in improving the quality of maternal and newborn care around childbirth (2). This paper addresses six of the eight standards, excluding standards 2 and 3 (Figure 1). The postintervention results will be reported in a future publication.

## Materials and methods

### Study design

Qualitative and quantitative methods were both used to assess the baseline implementation of quality-of-care interventions around childbirth at an intermediate hospital in northeast Namibia. We applied mixed-methods data collection as it aligns with the Donabedian and WHO frameworks for assessing quality-of-care facility. Also, the frameworks best suit our study as they are modeled to tell a story on care provision through the three components of care. The components include inputs, outputs/processes, and outcomes around childbirth. The qualitative data were collected by observing women in the maternity ward as they navigated admission, labor, and childbirth. In contrast, quantitative data assessed facility functionality and readiness, record review, and structured interviews with women discharged after delivery, staff, and the facility manager.

The research was supported by the Namibian Ministry of Health and the University of the Western Cape (UWC). Ethical approval was obtained from UWC and the Namibian Ministry of Health.

### Sampling

Kavango region, northeast Namibia, was purposively sampled because it has the only intermediate-referral hospital in that region. The hospital accounts for half of all deliveries in the region and has a high neonatal mortality rate (27/1,000) above the national level (20/1,000) (16). The factors that influenced the selection of the hospital included (1) high case load/deliveries, (2) poor newborn health indicators, and (3) being a UNICEF-supported region/hospital for maternal newborn programs. Also, the region records 72.8% health facility deliveries, 75% deliveries by skilled birth attendants, and 47.7% postnatal care within 2 days (16). Meanwhile, northeast Namibia's intermediate hospital deliveries increased from 8,823 in 2019 to 11,967 by 2020. By the time of data collection, infrastructure and human resources for health (17) were inadequate to accommodate the increasing deliveries, posing a challenge to the healthcare system, which is expected to improve quality healthcare amidst an overcrowded maternity unit. Yet, no quality improvement program existed.

The selection of staff for the interview (*N* = 20) was purposeful. The selection criteria included staff working with pregnant women, in the labor and delivery unit, and in the postnatal care and premature unit. The facility manager was conveniently selected for the interview as the only manager for the facility. Observed women (*N* = 53) were conveniently sampled as they were admitted in the maternity ward for labor and delivery during the data collection period. The women who delivered (*N* = 100) were also sampled conveniently for the interview during data collection when they were discharged home. The sampled numbers of the facility manager, staff, and observed and interviewed women were based on the estimated good reach on saturation and obtaining adequate voice representation. The woman was counted as part of the 53 if she was observed but did not completed four stages of childbirth. The stages included are as follows: admission into the maternity ward, labor, delivery, and immediate care after birth on the day of data collection. Of 53 observed women, 19 women completed the four stages.

For the record review, we purposively chose January to December 2016 and systematically sampled every 22nd delivery until the necessary sample size was reached. The calculated sample size was per the study protocol using 5,716 deliveries in 2016. With 0.05 alpha and 0.80 power, we needed a sample size of 211 before and after groups. So, for a full review of records as part of this baseline study (before the group), considering potential information in the records, we indicated reviewing 250 records of mother–newborn pairs. Thus, because of missing records, we reviewed 259 mother–baby pairs. The endline paper will report the results of the pre- and postintervention phases.

### Structure of the data collection tool

The EMEN tool is divided into six tools or forms. The facility’s structural and functionality readiness form1 assesses physical resources, supplies, equipment, and medicine. The management interview form2 assesses the policy environment, while form3 assesses the formal and refresher training the staff received in maternal and newborn care. The form also has vignettes to test staff knowledge of the subject areas. Form4 observes the women from admission to labor and delivery as she navigates the process of care. Form5 captures data on the care provided from the medical record. The form also collects outcome data and reviews partographs and records of women who underwent a cesarean section to deliver. Form6 assesses women's perceptions of the quality of care they received during hospitalization (Supplementary Table S3).

The EMEN assessment tool was developed by pulling together the best interventions of WHO’s Service Availability Readiness Assessment (SARA) and those used in vigorous research settings (9). By using the tool to collect data, we were able to capture gaps in quality of care identified in other large studies (9, 18–20) and across the WHO/UNICEF quality framework (Supplementary Figure S1 and Table S3). This demonstrates the strong validity and reliability of the EMEN tool and the results of this study. Since no single tool is sufficient to capture all quality measures (21–23), we encourage researchers to use a mixture of tools to derive the best benefit from the results. Even if it is one quality domain to be assessed, we used at least 3–4 EMEN tools to capture quality standards widely (Supplementary Table S2). Despite the EMEN tool having found a high implementation of human, essential physical resources, and drugs, we observed a few inconsistencies on the ground vs. the findings.

### Data collection

Assistant data collectors comprised one retired nurse and two nursing students who interviewed staff and reviewed maternity records. The data collectors also included two student doctors who conducted observations and exit interviews. The first author interviewed medical doctors. We collected data by adapting the Every Mother Every Newborn (EMEN) assessment tool into local context. The EMEN tool assesses the quality-of-care interventions during childbirth, especially the first 24 h (24). EMEN tool development was based on harmonizing interventions from tool(s) of WHO's SARA and those used in robust research settings (9). The final version incorporated experiences from implementing the same tool in Bangladesh, Ghana, and Tanzania. The assistant data collectors were trained by the UNICEF international consultant who led cross-sectional studies using similar study tools in the three countries. The training included observing them in practice, ensuring data quality and consistency. The EMEN tool has strong validity and reliability as it incorporates experiences from large-scale studies and robust surveys (9). Our other paper that assessed the capacity of the EMEN tool found it strong in capturing WHO/UNICEF/UNFPA maternal and newborn quality standards (15). The collected data did not include any respondents’ personal identifiers. Prior to each interview, the assessors read the oral consent script and asked the participant to respond “yes” or “no.” The interview proceeded with only those who consented. The data collection was from December 10, 2019 to January 19, 2020.

### Data analysis and management

Quantitative data were entered, coded, cleaned, and analyzed using SPSS for Mac, version 27. We used descriptive statistics to summarize key results into tables and figures. Since it was one site, the facility’s structural and functionality readiness and manager questionnaires were manually analyzed. We applied all six EMEN assessment tools to capture quality-of-care interventions around childbirth. We adopted the scoring analysis approach of the tools from Brizuela et al. (22). We found the approach useful and built on it to analyze data from the EMEN tool by benchmarking our results/responses captured by the tool against each quality measure (Supplementary Table S2). We expanded on the Brizuela et al. (22) scoring approach for assessing the capacity of tools to capture quality standard measures. In addition, instead of just reporting the number of quality items/questions present, we analyzed the proportion of responses from each tool against a WHO/UNICEF/UNFPA standard measure (Supplementary Table S2).

All the questions in the tools included measures related to inputs/processes/outputs/outcomes. We reviewed each questionnaire and matched questions in the tools with the WHO/UNICEF/UNFPA quality measures associated with the standards. A detailed description of the mapping exercise is published in our other paper (15). In summary, we matched questions/responses in the tools to each of the measures, which required warranting that all responses/questions in the tools and all measures were considered. For instance, responses on the availability of lifesaving supplies and functioning equipment for emergency care and newborn resuscitation were captured under facility readiness and observation of care tools.

For these analyses, we used descriptive statistics to calculate the average or proportion of responses captured by each tool. For quality measures with multiple subcomponents/questions, at least one of the subcomponents captured was considered enough. For example, a quality measure might list several medicines and the tool might measure a subset of the medicines on the list unless the quality measures clearly require that all subcomponents be present for the measure to be met (e.g., provision of essential newborn care required four elements, and the tools had to capture responses for all four). Then, we calculated the average or response percentage of quality measures captured per tool (e.g., the average response proportion of quality measures of a given quality statement captured within a specific tool) (Supplementary Table S2).

This was a crucial step in having a summarized table of results depicting clearly which indicator(s) or quality intervention(s) were poorly, moderately, or highly practiced. It then becomes easier to tell from the table (Supplementary Table S2) which EMEN tool captured most of the WHO/UNICEF/UNFPA quality measures under each quality statement and/or standard.

Data management for data collected around childbirth was performed using paper-based tools. The principal investigator checked the first 10 responses of each tool for completeness and consistency of codes. Since the principal investigator was on site, forms with identified problems were immediately given back to the assessor for verification and correction. The data were declared as a missing value if it could not be corrected using a register or the mother was not present at the time of verification. All completed clean data were handed over to the principal investigator for safekeeping. Only the data management team had access to the data. The data were entered into SPSS software by a statistician from Namibia University of Science and Technology, who, after entry, handed back all the records to the principal investigator for safekeeping and storage. The first author performed data cleaning before analysis.

## Results

This is Namibia's first study to assess, benchmark, and report the preliminary implementation of quality care interventions around childbirth against WHO /UNICEF/UNFPA quality standards. Also, this is the first study in Namibia to use the EMEN tool in assessing quality care around childbirth.

**Figure 2:**
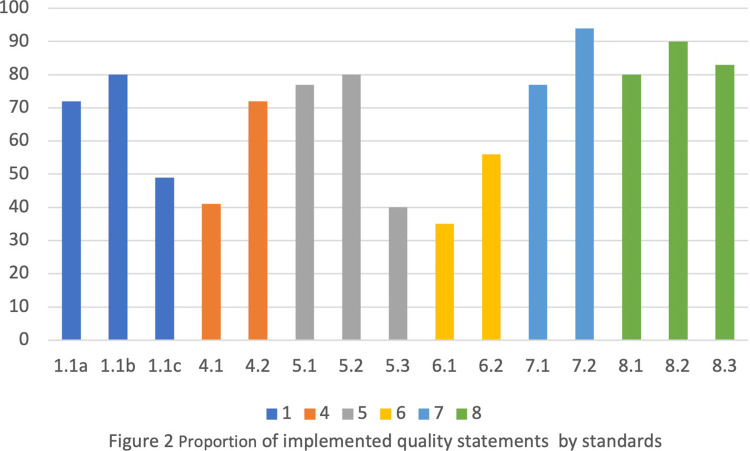
Proportion of implemented quality statements by standards.

**Figure 3:**
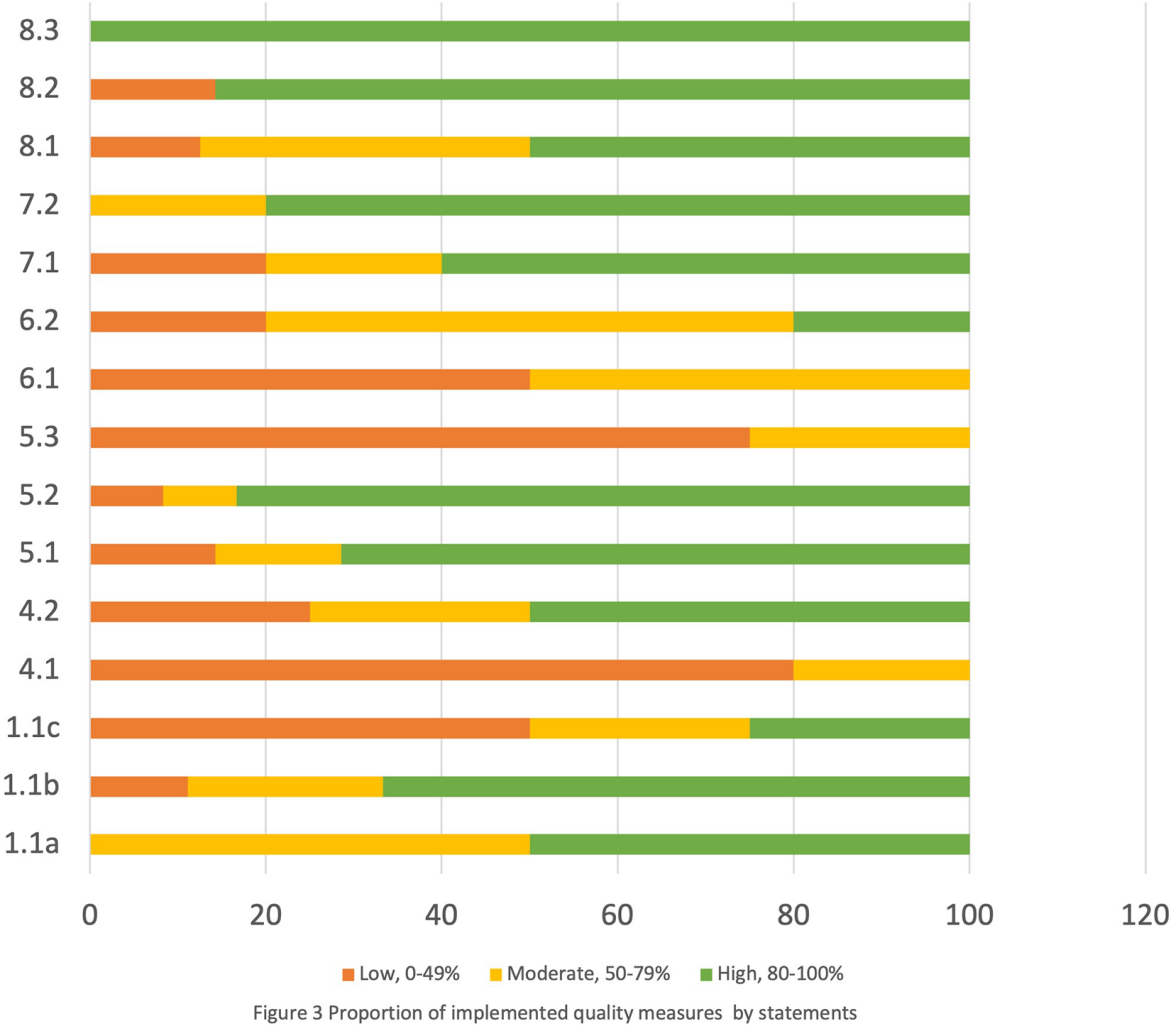
Proportion of implemented quality measures by statements.

A total of 259 maternity records were reviewed, and 100 women who had delivered, 20 staff, and a manager were interviewed. Another 53 were conveniently sampled women observed at initial presentation in the facility or at admission. While during the assessor's particular time in the ward, 19 women of 53 were observed to go through all stages of childbirth. The stages include admission, labor, delivery, and immediate care after birth. Thirty-four women were not observed for all stages of childbirth because those stages occurred outside the assessors’ time in the ward. A total of 53 observed and 100 interviewed women were decided as enough figures to obtain good saturation (25), representative opinions for women, and analysis power. This study defines the proportion of quality measures (Figures 2, 3) implemented as follows: low if responses were 0%–49% (red), moderate if responses were 50%–79% (yellow), and high if responses were 80%–100% (green).

Across the standards, there was above 50% implementation of quality intervention measures for admission, labor, and delivery. Also, 80% of essential physical resources were availabile, e.g., drugs, supplies, equipment, and sanitation facilities (Supplementary Table S2). Meanwhile, four quality statement measures (Supplementary Table S2) in standards 1, 4, 5, and 6 were found to be poorly implemented. The standards include evidence-based care, communication with women, respect and dignity, and emotional support. The poorly performed statement measures were on (1) newborn routine postnatal care (49%), (2) women receiving information on care and effective interactions with staff (41%), (3) women making informed choices on services they receive and are informed about interventions (40%), and (4) companion of choice (35%).

### Sociodemographic characteristics

Table 1 shows the socioeconomic demographic characteristic of women. In Table 1, 100 women who had delivered were interviewed at discharge to capture their opinions on the care they received around childbirth in the hospital. Adolescents aged 12–19 years were 36%, and those aged 20–24 accounted for 26%. Table 1 also depicts 27% (69 of 259) of the reviewed records as deliveries among adolescents (12–19 years). Most (41%) women were pregnant for the first time. Most (92%) women were unemployed (Table 1). According to the record review, 72% (187 of 259) of the women were unemployed.

**Table 1:** Women’s demographic characteristics.

Characteristic	Number of responses (%)	
	Mother interview (*N* = 100)	Record review (*N* = 259)
	*N* (%)	*N* (%)
Age		
<20 years	36 (36%)	69 (27%)
20–24 years	26 (26%)	60 (23%)
25–29 years	20 (20%)	55 (21%)
30+ years	18 (18%)	70 (27%)
Not recorded	n/a	05 (2%)
Education		
None	**2** **(2%)**	**0** **(0%)**
Primary	24 (24%)	5 (2%)
Secondary	68 (68%)	5 (2%)
Tertiary	6 (6%)	4 (2%)
Not recorded	n/a	245 (95%)
Employment		
Employed	8 (8%)	29 (11%)
Unemployed	92 (92%)	187 (72%)
Not recorded	n/a	43 (17%)

### Healthcare at admission and during labor and childbirth: standard 1

Table 2 shows above 50% implementation of various quality intervention measures, statement 1.1a, meaning that women are assessed routinely at admission and during labor and childbirth and measures were implemented (Table 2) for admission, labor, and childbirth. For example, most women (range 75%–98%) reported receiving and/or being provided with routine examinations and checkups for key maternal and newborn care parameters. The critical vital signs checked included blood pressure measurement (90%), checking for fetal heart rate (92%), and testing women's urine (75%) for proteins. Further, assistant data collectors observed a11.2% of women whose labor was monitored using a partograph (Table 2). Oxytocin for Active Management of the Third Stage of Labour (AMTSL) is among the critical indicators for childbirth and was administered to 84.2% of women. Supplementary Table S2 shows that 40% of the interviewed staff received training/refresher in obstetrics, newborn care, and breastfeeding in the past 12 months (Supplementary Table S1). Also, on average, 80% of newborns received the WHO/UNICEF/UNFPA interventions for quality statement1.1b measures on care immediately after birth (Supplementary Table S2). For example, more than 85% of babies were observed receiving all four elements of essential newborn care. The elements include immediate thorough drying, immediate skin-to-skin contact, delayed cord clamping, and initiation of breastfeeding in the first hour (Supplementary Table S2).

**Table 2:** Proportion of women assessment report, staff–client interactions, and overall satisfaction with care.

Aspect of care	Modality for which women's and assessors’ perspective around childbirth interventions was assessed	Number of responses (%)	
		Women's report (*N* = 100)	Assessors’ observations (*N* = 53)
Vital signs checked at admission	Eyelids/tongue/nails	26 (26%)	1 (2%)
	Blood pressure	90 (90%)	52 (98%)
	Urine tested (*N* = 52, 1 = N/A)	75 (75%)	41 (79%)
	Fetal heart rate	92 (92%)	50 (94%)
Provider–client interactions	Examination feedback given	47 (47%)	14 (27%)
	Had an opportunity to discuss concerns	38 (38%)	
	Informed about actions and procedures taken, e.g., C/S, plan for delivery	17 (17%)	1 (2%)
	Responsiveness of the health care provider (HCP)	81 (81%)	
Satisfaction with care	Satisfied with healthcare services	69 (69%)	
	Satisfied with information received from providers on breastfeeding	48 (48%)	
	Satisfied with information received from providers on postpartum care	41 (41%)	
	Satisfied with information received from providers on family planning	28 (28%)	
	Satisfactory attitude of the HCP	87 (87%)	
	Level of attention given to the baby	98 (98%)	
	Recommend this facility to others	91 (91%)	
	Will return to the same facility	82 (82%)	

### Healthcare for routine postnatal newborn care: standard 1

Overall, Supplementary Table S2 shows that less than 50% of the newborns received routine postnatal care interventions as per WHO/UNICEF/UNFPA quality statement 1.1c. For instance, although 100% (19 of 19) babies were observed being examined and receiving vitamin K and full immunization immediately after birth and before being discharged home, the proportion of babies examined before discharge reduced to 62%. In contrast, less than 50% of women reported receiving breastfeeding, postpartum hygiene, and family planning information from providers. Providers counseling women on maternal and newborn danger signs and when to seek immediate care from the nearest health facility was low and rarely practiced (Supplementary Table S2).

### Healthcare on provider–client interaction/experience of care: standards 4, 5, and 6

Figures 2 and 3 and Supplementary Table S2 show less than 50% implementation of WHO/UNICEF/UNFPA three quality statements on interventions/measures within the experience of care domain standards. They include standards 4, 5, and 6 on communication with women, respect and dignity, and emotional support, respectively (Figures 2, 3). While within the three standards, one quality statement measure was poorly practiced (range 35%–41%). The poorly implemented quality statements include statement 4.1 (women and their families receive information about the care and have effective interactions with staff); statement 5.3 (all women make informed choices about the services and interventions they receive and interventions are explained to them); and statement 6.1 (every woman is offered the option to experience labor and childbirth with a companion) (Figures 2 and 3 and Supplementary Table S2).

Figures 2 and 3 and Supplementary Table S2 also show that 69%–84% of women reported satisfaction with the health services and nurses’ attitude and felt their privacy was maintained during examinations and provider–client interactions. Also, recommending the same facility to others and themselves returning to the same facility for delivery were reported by 91% and 84% of women, respectively. On the contrary, more than half of the women reported not receiving feedback postexamination. Neither were they given the opportunity to express their concerns. Also, according to the assessors’ observations, 98% of women were not informed of the delivery plan. Also, Supplementary Table S2 shows that women were rarely allowed a companion of choice during delivery.

### Human and essential physical resources for healthcare: standards 7 and 8

Supplementary Table S2 shows that although human resources for health were found universally available, less than half of them were trained/refreshed in critical maternal and newborn skills. The skills include early postnatal care and breastfeeding (Supplementary Table S2). On average, 80%–90% of essential physical resources, e.g., drugs, supplies, and equipment, were available in adequate amounts (Supplementary Table S2). They include magnesium sulfate for managing severe pre-eclampsia and oxytocin for Active Management of the Third Stage of Labour (AMTSL) according to WHO guidelines.

## Discussion

### Summary of healthcare gaps

This is the first baseline study in northeast Namibia to assess the implementation of WHO/UNICEF/UNFPA standards for improving the quality of maternal and newborn care around childbirth. The study assessed WHO/UNICEF/UNFPA standards and quality measures for maternal and newborn care around childbirth using the Donabedian and WHO frameworks. This study is among the few that identified noteworthy gaps across the three WHO quality-of-care domains. The poor and inconsistent implementation of the communication measures within the experiences of care domain is intertwined with other standards and affects other domains. This resulted in low/poor postpartum and postnatal newborn care within evidence-based care standard 1 or the first domain, while the second domain includes low/poor provider–client information sharing or communication and low/poor women involvement in decisions and actions taken about their care. Our results are similar to the findings from past studies (9, 18–20, 26) conducted in Bangladesh, Ghana, Tanzania, Kenya, and India that used mixed methods and direct observation.

### Quality of healthcare during labor and delivery/around birth

Evidence that essential supplies, medicines, equipment, and evidence-based clinical practices are in place is a key quality-of-care function or element (10, 27). In this study, availability of essential physical resources, supplies, medicines, and sanitation facilities was high. For instance, there was a good stock of magnesium sulfate for the management of Pre-eclampsia and hypertensive disorders and oxytocin for postpartum hemorrhage and Active Management of the Third Stage of Labour. Although our results were based on one high-volume site, our result was inconsistent with findings from Bangladesh (19), where the availability of magnesium sulfate and oxytocin was 13.3% (2 of 15) and 6.7% (1 of 15), respectively (19). In this study, none of the women purchased any supplies including drugs. This result was again contrary to the findings from Bangladesh (19), where 83% of administered oxytocin for AMTSL was for women who self-purchased from private pharmacies.

Abdominal examination, monitoring of fetal heart rate, and vaginal examination at regular intervals can facilitate early identification of labor complications and timely management. WHO states the importance of blood pressure measurement and urine testing in detecting pre-eclampsia (28). In this study, blood pressure was measured for more than 90% of women and urine was checked for proteins of 75% of women. This result contradicts a Bangladesh study, where only 50% of women were checked for blood pressure and rare urine testing (19). Partograph monitoring is a key WHO-recommended early warning tool designed to help monitor the progress of labor activities (29). Meanwhile, in this study, only very few women's labor was monitored using a partograph. This result is not impressive but still contrary to one study where none of the assessed facilities used a partograph (19). In Namibia, partograph monitoring is part of the labor care guide and the recently revised maternity records. However, this finding suggests that birth attendants are noncompliant with existing guides and protocols. Thus, staff working with pregnant women and in labor and delivery areas should be regularly supplied with partographs, trained/refreshed (27) on their proper use and timely documentation of vital signs, and supervised for improved labor outcomes (19).

### Quality of healthcare in the postnatal period

Another gap was identified beyond labor and delivery and was present in immediate postnatal newborn care. Consistent with studies in Bangladesh (19) and India (30), immediately after birth, most neonates received all four elements of essential newborn care. Also, all newborns were examined and received vitamin K and full immunization. Yet, almost 40% of newborns were discharged home without being examined. In Namibia, most discharges for normal deliveries happen within 24–72 h, implying that most newborns who develop complications or conditions within 72 h postdelivery are discharged home unidentified or undiagnosed. These nonexamined newborns at discharge may go home with a severe condition(s) or danger sign(s). Depending on the mother's level of education on danger signs and how far they live from the nearest health facility, newborns may be at risk of preventable deaths due to delay in seeking care or late identification of the condition. This result is consistent with an Indian study where few newborns were examined in the postnatal ward (18).

Despite WHO recommending the provision of postnatal care for both the mother and baby (5), that it has a protective effect on neonatal death outcomes (31) and that postnatal care is an opportune time to provide care that prevents maternal and newborn deaths (30) there was a minimal implementation of WHO/UNICEF/UNFPA recommended standard interventions for postnatal newborn care, confirming the vulnerability of the neonates and their mothers around childbirth, immediately after birth, or in the postnatal period (32–35). The Namibia DHS reports similar low maternal postnatal care coverage within 48 h postdelivery (16), suggesting and confirming that the training provided to providers since then has not translated into improved actions around childbirth and immediate postnatal care.

Another gap identified within postnatal care is providers’ inadequate knowledge and skills in managing maternal and newborn complications including sick newborns. During labor, childbirth, and postnatal care, midwives play a crucial role in saving lives and preventing physical and psychological morbidities (3). Providers are also in constant direct contact with mothers and newborns (36). Yet, most staff in this study and other settings lack knowledge and skills in postnatal care (30) and breastfeeding management (3, 37), implying that only a few staff members were capable providers. Thus, without providers’ regular training/refreshers on maternal and newborn care, quality care around birth will not improve. Our next paper linked to this study reports on causes of newborn deaths after quality improvement interventions.

### Quality-of-care standards on provider–client interactions/experience of care

Another key gap was the low implementation of WHO/UNICEF/UNFPA quality intervention measures related to staff–client information sharing, involvement, and interactions. Capturing women's voices or client's perception of health services is critical for quality improvement (22, 38). Similarly, communicating with women and involving them in their and newborns’ care, alleviate anxiety, enabling them to make informed choices, which increases compliance and satisfaction with care (5, 39). Yet, in this study, providers minimally gave feedback to women on the assessments done and care actions taken. As reported by Erchafo and others (40), less involvement of women in their and their babies’ care can make them feel less valued, disrespected, and mistreated. Another implication of women not being aware of procedures or actions taken for their care is that it can affect their health beyond labor and childbirth (41). The implications have immediate and long-term effects on women. The effects include long-term negative childbirth experiences, e.g., post-traumatic disorder, and persistent fear of childbirth (41). Other effects include women's decisions to seek care, fearing mistreatment and inappropriate care (39) due to previous negative birth experiences, poor healthcare, and neglect (42). In this study, facility deliveries are high. Thus, continued providers’ actions of not involving women in their and newborns’ care may reduce future facility deliveries based on negative birth experiences. When this happens, women may opt for home deliveries, which can increase their and their newborns’ vulnerability, Implying that to improve quality care practices around childbirth, training of all personnel in interpersonal communication and provider–client information sharing needs prioritization. These poor care practices around childbirth are consistent with past studies (18, 19, 43), including information sharing on postpartum care (18). Similar to reports from previous studies (14, 18, 44), informing women about maternal and newborn danger signs and when to seek care was minimal, suggesting that many women delay timely careseeking for themselves and newborns due to a lack of knowledge of danger signs. Healthcare seeking can also be delayed when women and their families do not understand the implications of late careseeking if a danger sign is present. Our results confirm the argument that creating two-way communication with mothers and involving them in the care are abandoned elements of quality of care (18). Thus, improving measures around childbirth will be difficult without improving staff–client communication across three quality-of-care domains.

Companion of choice is another experience of the care gap. Experiencing labor and childbirth with a companion of choice has been reported to aid women's positive birth experience (45) and improve the birth outcome (5, 46). Further, allowing a companion during labor and delivery is associated with respectful maternity care (47, 48) and quality-of-care provision (45). According to women in Italy (14), allowing a companion can contribute to improved quality care. Yet, in this study, none of the women experienced birth with a companion of choice (Supplementary Table S2), although 69% of women in this study were accompanied to the facility for delivery. The opportunity to allow women the same companion throughout labor and childbirth was missed. Our result is consistent with studies in Kenya (26), Malawi (39), Ghana, and Tanzania (20) but contrary to findings from a study in Bangladesh (20). Factors hindering companionship implementation include the lack of privacy and space and healthcare workers and women not recognizing the benefits associated with birth companions (46). We did not explore reasons for very low companionship practices as it was beyond the study scope. Research is needed to investigate reasons for low companionship practices and their impact on birth outcomes and quality-care implications in the Namibian context.

### Contextual measurement of the WHO/UNICEF/UNFPA quality standards

Overall, the WHO/UNICEF/UNFPA maternal and newborn quality improvement standards are comprehensive in nature as they cover all three (input/process/outcomes) components of care. We found their implementation useful in depicting quality-of-care strengths and gaps around childbirth. We agree with previous authors (47) that clear guidance and recommendations on how to operationalize and rank the measures are critically needed. During our data collection, we noted context-specific comments for possible consideration in a future revision of indicators. Annotation of some indicators, e.g., optional, can assist countries during implementation (for a few examples, see Supplementary Table S4).

### Implications for the healthcare system and policy

The Namibia health system is faced with a litmus test to provide healthcare improvement in response to the increasing number of newborn deaths amidst increasing facility deliveries. In previous studies that assessed similar implementation of WHO/UNICEF/UNFPA standards (49), a significant reduction of preventable maternal, perinatal, and newborn deaths was recorded. Supplementary Table S5 summarizes key good interventions and areas of concern noted during the interviews and observation of quality-of-care standards around childbirth. Our results are informing UNICEF's current interventions support to improve quality-of-care beyond the study site. The support focuses on maternity settings and neonatal care units where most of the newborn deaths occur. The first author advocated for quality improvement beyond the study site at the initial protocol development or data collection stage. The purpose of the advocacy is to inform the use of baseline results and recommendations beyond the study site for sustained ownership. However, this requires strong health system management and enabling policy environment across the three quality-of-care domains around childbirth.

### Strengths and limitations

This study is not without limitations. As mentioned by previous authors (15, 22, 23), WHO standards present a good direction, but the challenge remains with the absence of standard criteria for assessing quality care, particularly in low- and middle-income countries. Another limitation common with observational and facility interviews is that staff actions may have been altered due to the presence of the assessors (Hawthorn effect), although their presence in the facility over 2 weeks could potentially have minimized the Hawthorn effect. This study report on the quality-of-care standards around childbirth. Meanwhile, the rationale for minimal companionship and decrease in care immediately postnatally/after birth was beyond the study scope. Direct observation of services is reported to be a gold standard for assessing any component of health or activity (38). Our study assessed the quality-of-care standards around childbirth at admission, in labor and delivery, and immediately after birth to the time of discharge through observation. The use of data collection tools tested in large studies (9, 20, 49) and its capacity peer-reviewed (15) for assessing quality-of-care standards around childbirth was also a strength.

## Conclusion

To the best of our knowledge, this is the first study in Namibia to assess the implementation of the WHO/UNICEF/UNFPA quality-of-care measures around childbirth. The measures of provider–client interactions and information sharing identified significant deficiencies in this aspect of care that negatively affected the client's experience of care. To achieve reductions in neonatal death, improved training in communication skills to educate clients is likely to have a major positive and relatively low-cost impact.

The foundation to build quality improvement programs existed in terms of essential physical resources, clinical care processes, policies, guidelines, and human resources. Several gaps were identified that can inform health system priorities to strengthen maternal and newborn quality improvement around childbirth, particularly improving immediate postpartum and postnatal newborn care, involving women in decisions and actions taken about their and neonates’ care, and staff–client interpersonal communication. However, it is humbling to witness how Namibia has invested in the healthcare system, signaling its commitment to improving quality care in public health facilities. More women are coming to deliver at facilities, but the question remains whether the healthcare system staff and space capacity are ready to manage the increasing demand, suggesting that healthcare system managers need to pay attention and address gaps hindering quality care provision around childbirth. Data gathered in this study were useful in informing the current design and implementation of the quality improvement program beyond the study site. The results of this study can benefit and contribute to a future revision of the WHO/UNICEF/UNFPA standards. The standards are interwoven and complement each other. Further WHO guidance is needed for LMICs on standard context criteria for assessing the WHO/UNICEF/UNFPA quality care measures around childbirth.

## Data Availability

The original contributions presented in the study are included in the article/**Supplementary Material**, further inquiries can be directed to the corresponding author/s.
