# Supplementary material for: Baseline assessment of the WHO/UNICEF/UNFPA maternal and newborn quality-of-care standards around childbirth: Results from an intermediate hospital, northeast Namibia
Source: Front Pediatr. 2023 Jan 9;10:972815. doi: 10.3389/fped.2022.972815 (PMC9869061; doi:10.3389/fped.2022.972815)
Supplement: Supplementary file 1 [file Datasheet1.pdf]

S1 Table WHO/UNICEF/UNFPA Standards of care and quality statements
Standard 1: Every woman and newborn receive routine, evidence-based care and management of complications during labour, childbirth and the early postnatal period, according to WHO guidelines.
Quality statements
1.1a: Women are assessed routinely on admission and during labour and childbirth and are given timely, appropriate care.
1.1b: Newborns receive routine care immediately after birth.
1.1c: Mothers and newborns receive routine postnatal care.
1.2: Women with pre-eclampsia or eclampsia promptly receive appropriate interventions, according to WHO guidelines.
1.3: Women with postpartum haemorrhage promptly receive appropriate interventions, according to WHO guidelines.
1.4: Women with delay in labour or whose labour is obstructed receive appropriate interventions, according to WHO guidelines.
1.5: Newborns who are not breathing spontaneously receive appropriate stimulation and resuscitation with a bag-and-mask within 1 min of birth, according to WHO guidelines.
1.6a: Women in preterm labour receive appropriate interventions for both themselves and their babies, according to WHO guidelines.
1.6b: Preterm and small babies receive appropriate care, according to WHO guidelines.
1.7a: Women with or at risk for infection during labour, childbirth or the early postnatal period promptly receive appropriate interventions, according to WHO guidelines.
1.7b: Newborns with suspected infection or risk factors for infection are promptly given antibiotic treatment, according to WHO guidelines.

1.8: All women and newborns receive care according to standard precautions for preventing hospital-acquired infections.
1.9: No woman or newborn is subjected to unnecessary or harmful practices during labour, childbirth and the early postnatal period.
Standard 2: The health information system enables use of data to ensure early, appropriate action to improve the care of every woman and newborn.
Quality statements
2.1: Every woman and newborn has a complete, accurate, standardized medical record during labour, childbirth and the early postnatal period.
2.2: Every health facility has a mechanism for data collection, analysis and feedback as part of its activities for monitoring and improving performance around the time of childbirth.
Standard 3: Every woman and newborn with condition(s) that cannot be dealt with effectively with the available resources is appropriately referred.
Quality statements
3.1: Every woman and newborn is appropriately assessed on admission, during labour and in the early postnatal period to determine whether referral is required, and the decision to refer is made without delay.
3.2: For every woman and newborn who requires referral, the referral follows a pre-established plan that can be implemented without delay at any time.
3.3: For every woman and newborn referred within or between health facilities, there is appropriate information exchange and feedback to relevant health care staff.
Standard 4: Communication with women and their families is effective and responds to their needs and preferences.
Quality statements
4.1: All women and their families receive information about the care and have effective interactions with staff.
4.2: All women and their families experience coordinated care, with clear, accurate information exchange between relevant health and social care professionals.
Standard 5: Women and newborns receive care with respect and preservation of their dignity.
Quality statements
5.1: All women and newborns have privacy around the time of labour and childbirth, and their confidentiality is respected

5.2: No woman or newborn is subjected to mistreatment, such as physical, sexual or verbal abuse, discrimination, neglect, detainment, extortion or denial of services.
5.3: All women have informed choices in the services they receive, and the reasons for interventions or outcomes are clearly explained.
Standard 6: Every woman and her family are provided with emotional support that is sensitive to their needs and strengthens the woman’s capability.
Quality statements
6.1: Every woman is offered the option to experience labour and childbirth with the companion of her choice.
6.2: Every woman receives support to strengthens her capability during childbirth.
Standard 7: For every woman and newborn, competent, motivated staff are consistently available to provide routine care and manage complications.
Quality statements
7.1: Every woman and child has access at all times to at least one skilled birth attendant and support staff for routine care and management of complications.
7.2: The skilled birth attendants and support staff have appropriate competence and skills mix to meet the requirements of labour, childbirth and the early postnatal period.
7.3: Every health facility has managerial and clinical leadership that is collectively responsible for developing and implementing appropriate policies and fosters an environment that supports facility staff in continuous quality improvement.
Standard 8: The health facility has an appropriate physical environment, with adequate water, sanitation and energy supplies, medicines, supplies and equipment for routine maternal and newborn care and management of complications.
Quality statements
8.1: Water, energy, sanitation, hand hygiene and waste disposal facilities are functional, reliable, safe and sufficient to meet the needs of staff, women and their families.
8.2: Areas for labour, childbirth and postnatal care are designed, organized and maintained so that every woman and newborn can be cared for according to their needs in private, to facilitate the continuity of care.
8.3: An adequate stock of medicines, supplies and equipment is available for routine care and management of complications.
