# Supplementary material for: Baseline assessment of the WHO/UNICEF/UNFPA maternal and newborn quality-of-care standards around childbirth: Results from an intermediate hospital, northeast Namibia
Source: Front Pediatr. 2023 Jan 9;10:972815. doi: 10.3389/fped.2022.972815 (PMC9869061; doi:10.3389/fped.2022.972815)
Supplement: Supplementary file 2 [file Datasheet2.pdf]

S2 Table. EMEN tool responses (%) against WHO/UNICEF/UNFPA quality standards

	Proportion of quality measures implemented*						
	Form1	Form2	Form3	Form4	Form5	Form6	Average score
Standard 1: Every woman and newborn receives routine, evidence-based care, and management of complications during labour, childbirth, and the early postnatal period, according to WHO guidelines.							
Quality statement 1.1a: Women are assessed routinely on admission and during labour and childbirth and are given timely, appropriate care							
Quality measures							
Input quality measures							
1.The facility has the basic essential equipment and supplies for routine care and detection of complications (thermometers, sphygmomanometer, fetal stethoscopes, urine dipsticks) available in sufficient quantities at all times in the areas of the maternity unit for labour and childbirth.	100			88		83	90
3.The health care staff in the labour and childbirth areas of the maternity unit receive in-service training and regular refresher sessions at least once every 12 months in the identification and management of obstetric emergencies during labour and childbirth			40				40
Output/process quality measures							
1.The proportion of all women who gave birth in the health facility whose blood pressure, pulse, and temperature were appropriately recorded during labour, childbirth, and the early postnatal period (and acted on if appropriate)				98		90	94
2.The proportion of all women who gave birth in the health facility who received oxytocin within 1 min of the birth of their baby.				84	89		87
4.The proportion of all women who gave birth in the health facility whose urinalysis result was appropriately recorded during labour, childbirth, and the early postpartum period (and acted on if appropriate).				77		75	76
5.The proportion of all women who gave birth in the health facility who received any option for pain relief during labour and childbirth.				63		25	44
Average score for quality statement 1.1a							72
Quality statement 1.1b. Newborn receive routine care immediately after birth							
Input quality measures							
1.The health facility has written, up-to-date, clinical protocols for essential newborn care that are consistent with WHO guidelines and are available in the labour and childbirth areas of the maternity unit.		100					100
2.The health facility has supplies of sterile cord ties (or clamps) and scissors (or blades), available in sufficient quantities at all times for the expected number of births	100			95			98
3.The health facility has supplies of clean towels in the labour and childbirth areas for immediate drying of newborns, available in sufficient quantities at all times for the expected number of births				58			58

4.Health-care staff in the labour and childbirth areas of the maternity unit receive in-service training or regular refresher sessions in essential newborn care and breastfeeding support at least once every 12 months			40				40
Output/process quality measures							
1. The proportion of all newborns who were breastfed with 1 h of birth						57	57
2.The proportion of all newborns who were kept skins to skin contact (with body and head covered) with their mothers at least 1 h after birth			100	93		72	88
3.The proportion of all newborns who received all four elements of essential newborn care: immediate and thorough drying, immediate skin-to-skin contact, delayed cord clamping and initiation of breastfeeding in the first hour.			100	93		65	86
4. The proportion of all newborns whose umbilical cord was clamped 1-3 min after birth			100	90			95
5. The proportion of all newborn who were dried immediately and thoroughly at birth.			100	95			98
Average score quality statement 1.1b							80
Quality statement 1.1c Newborn receive routine postnatal care							
Input quality measures							
4.Health-care staff in the maternity unit receive in-service training and regular refresher sessions in routine postnatal care and breastfeeding at least every 12 months			45	5			25
6. The health facility has local arrangements for alternative feeding methods, including cup or cup-and spoon feeding, and avoids bottle-feeding	100		51				76
8. The health facility ensures that feeding of infant formula is demonstrated to mothers and family members of newborns only when needed, with a full explanation of the hazards of improper use.			53				53
Output/process quality measures							
1.The proportion of all newborns on postnatal care wards or areas in the health facility who received vitamin K and full vaccination as per national guidelines				100	87		94
4.The proportion of all newborns in the health facility who received a full clinical examination before discharge						62	62
7.The proportion of all healthy mothers on postnatal wards or areas in the health facility who received breastfeeding counselling and support from a skilled health care provider				5	90	48	48
9. The proportion of all postpartum women in the health facility who were offered counselling on birth spacing and family planning methods before discharge				5		28	17
Outcome quality measures							
2.The proportion of all postpartum women in the health facility who received contraception counselling on birth spacing and family planning before discharge.				5		28	17
Average score 1.1c newborn receive routine postnatal care							49
Standard 4: Communication with women and their families is effective and responds to their needs and preferences.							

Quality statement 4.1 All women and their families receive information about the care and have effective interactions with staff.							
Output/process quality measures							
1.The proportion of all women discharged from the labour and childbirth area of the facility who received written and verbal information and counselling on the following elements before discharge: nutrition and hygiene, birth spacing and family planning, exclusive breastfeeding and maintaining lactation, keeping their baby warm and clean, communication and play with the baby, danger signs for the mother and newborn and where to go in case of complications.				3	47	39	29
2.The proportion of all women who gave birth in the health facility who reported that they were given the opportunity to discuss their concerns and preferences.						38	38
Outcome quality measures							
1.The proportion of all women who gave birth in the health facility who felt they were adequately informed by the care provider(s) about the examinations, actions and decisions taken for their care.				14		42	28
3.The proportion of all women who gave birth in the health facility who expressed satisfaction with the health services.						69	69
4.The proportion of all women who gave birth in the health facility who reported that they were satisfied with the health education and information they received from care providers.						39	39
Average score for quality statement 4.1							41
Quality statement 4.2 All women and their families experience coordinated care, with clear, accurate information exchange between relevant health and social care professionals.							
Input quality measures							
1.The health facility has a standard form for clinical progress notes and monitoring events during labour (partograph), birth and after birth to facilitate written hand-over.			50	26			38
2.The health facility has written, up-to-date protocols for verbal and written hand-over of women and newborns at shift changes, during intra-facility transfer, on referral to other facilities and at discharge.		100	63				81
4.The health facility has a functioning, reliable communication system for information exchange among relevant services providers.	100	100					100
Outcome quality measures							
1.The proportion of all women who gave birth in the health facility who expressed satisfaction with the health services.						69	69
Average score for quality statement 4.2							72
Standard 5: Women and newborns receive care with respect and can maintain their dignity.							
Quality statement 5.1 All women and newborns have privacy around the time of labour and childbirth, and their confidentiality is respected.							
Input quality measures							

1.The physical environment of the health facility allows privacy and the provision of respectful, confidential care, including the availability of curtains, screens, partitions, and sufficient bed capacity.	100			83		84	89
2.The health facility has written, up-to-date protocols to ensure privacy and confidentiality for all women and newborns in all aspects of care.		100	100				100
3.The health facility has accountability mechanisms for redress in the event of violations of privacy, confidentiality, or consent.		100	69				85
Output/process quality measures							
2.The proportion of all women undergoing examinations or procedures in the health facility who reported that their permission was sought before the examination or procedures were performed.				14		42	28
Outcome quality measures							
1.The proportion of all women who gave birth in the health facility who were satisfied with the degree of privacy during their stay in the labour and childbirth areas.				83		83	83
2.The proportion of all women examined and treated in the health facility who expressed satisfaction with the degree of privacy during examinations and treatment.				83		84	83
3.The proportion of all women who gave birth in the health facility who expressed satisfaction with the health services.						69	69
Average score for quality measure 5.1							77
Quality statement 5.2 No woman or newborn is subjected to mistreatment, such as physical, sexual, or verbal abuse, discrimination, neglect, detainment, extortion, or denial of services.							
Input quality measures							
1.The health facility has written, up to date, zero-tolerance non-discriminatory policies with regard to mistreatment of women and newborns.		100	100				100
2.The health facility has a system whereby the mothers of small, sick newborns can be close to and nurse their babies.	100	100					100
3.The fee structures for maternity and newborn care are equitable, affordable, and clearly displayed.		100	100				100
4.The health facility has written accountability mechanisms for redress in the event of mistreatment.		100	100				100
5.The health facility has a written, up-to-date policy and protocols that outline women's and families' right to make a complaint about the care received and has an easily accessible mechanism (e.g., a box) for handing in complaints.	100	100	100				100
6.Health care staff in the maternity unit receive in-service training and supportive supervision in respecting the rights of mothers and newborns, respectful care and accountability mechanisms. Orientation is provided for new staff.		100	82				91
7.The health facility policy for payment specifically precludes detention of a woman or baby for non- payment.		100		100		100	100
8.The health facility has a complaints box, which is easily accessible to women and their families, is periodically emptied and the contents reviewed.	100						100
Output/process quality measures							

1.The proportion of all women who gave birth in the health facility who reported physical, verbal, or sexual abuse, to themselves or their newborns, during labour or childbirth or after birth.						16	16
3.The proportion of women who attended the health facility who were refused care because of their inability to pay.						0	0
Outcome quality measures							
1.The proportion of all women who gave birth in the health facility who expressed satisfaction with the health services.						69	69
2.The proportion of all women who gave birth in the health facility who reported having been treated with respect and their dignity preserved.						89	89
Average quality statement 5.2							80
Quality statement 5.3 All women can make informed choices about the services they receive, and the reasons for interventions or outcomes are clearly explained.							
Output/process quality measures							
2.The proportion of all women who gave birth in the health facility who felt adequately informed by health care staff regarding decisions taken about their care.				14		42	28
Outcome quality measures							
1.The proportion of women who gave birth in the health facility by caesarean section who were aware of the reason for the caesarean section.				2		73	38
2.The proportion of all women who gave birth in the health facility who expressed satisfaction with the health services.						69	69
3. The proportion of women who gave birth in the health facility who felt they had shared decisions about their labour, birth, and postnatal care				2		47	25
Average score quality statement 5.3							40
Standard 6: Every woman and her family are provided with emotional support that is sensitive to their needs and strengthens the woman's capability.							
Quality statement 6.1 Every woman is offered the option to experience labour and childbirth with the companion of her choice.							
Output/process quality measures							
1.The proportion of all women who gave birth in the health facility who had a companion of their choice during labour and childbirth.				0		2	1
Outcome quality measures							
1.The proportion of all women who gave birth in the health facility who expressed satisfaction with the health services.						69	69
Average score quality statement 6.1							35
Quality statement 6.2 Every woman receives support to strengthen her capability during childbirth.							
Input quality measures							

4.The health facility has a referral mechanism for women and families with complex emotional needs and refers them for specialist care.				60			60
Output/process quality measures							
1.The proportion of all women who gave birth in the health facility who did so in the labour position of their choice.				0			0
2.The proportion of all women undergoing bereavement or an adverse outcome who received additional emotional support from health facility staff.				60			60
Outcome quality measures							
1.The proportion of all women who gave birth in the health facility who expressed satisfaction with the health services.						69	69
2.The proportion of all women who gave birth in the health facility who would recommend childbirth in that facility.						91	91
Average score for quality statement 6.2							56
Standard 7: For every woman and newborn, competent, motivated staff are consistently available to provide routine care and manage complications.							
Quality statement 7.1 Every woman and child have access at all times to at least one skilled birth attendant and to support staff for routine care and management of complications.							
Input quality measures							
1.The health facility always has skilled birth attendants available, in sufficient numbers to meet the anticipated workload.	100	100					100
3.The health facility has a roster that is displayed in all areas, giving the names of staff on duty, the times of their shifts and their specific roles and responsibilities.		100					100
5.The health facility has clear communication channels to reach staff on duty at all times.	100	100					100
Outcome quality measures							
2.The proportion of all women who gave birth at the health facility who reported having been informed about danger signs for her and her baby and emergency preparedness.				5	8	29	14
3.The proportion of women who attended the health facility who were satisfied with the health care they received.						69	69
Average quality statement 7.1							77
Quality statement 7.2 The skilled birth attendants and support staff have appropriate competence and skills mix to meet the requirements of labour, childbirth, and the early postnatal period.							
Input quality measures							
1.The health facility has a programme for continuing professional development and skills development for all skilled birth attendants and other support staff and conducts regular training.		100	100				100
2.The health facility has standard procedures and plans for recruitment, deployment, motivation (recognition and reward scheme) and retention of all staff.		100					100

3.The health facility periodically appraises all staff and has a mechanism for recognizing good performance.		100					100
4.The health facility has sufficient numbers of educated, competent, licensed, motivated, regulated skilled birth attendants with an appropriate skills mix, working in multidisciplinary teams.	100	100					100
Outcome quality measures							
1.The proportion of all women who gave birth at the health facility who were satisfied with the care and support from facility staff.						69	69
Average quality statement 7.2							94
Standard 8: The health facility has an appropriate physical environment, with adequate water, sanitation and energy supplies, medicines, supplies, and equipment for routine maternal and newborn care and management of complications.							
Quality statement 8.1 Water, energy, sanitation, hand hygiene and waste disposal facilities are functioning, reliable, safe, and sufficient to meet the needs of staff, women, and their families.							
Input quality measures							
1.The health facility has a functioning source of safe water located on the premises that is adequate to meet all demands for drinking, personal hygiene, medical interventions, cleaning, laundry, and cooking for use by staff, women, newborns and their families.	100						100
2.The health facility has leak-proof, covered, labelled waste bins and impermeable sharps containers available in every treatment area, to allow segregation of waste into four categories: sharps, non- sharps infectious waste, general non-infectious waste (e.g., food, packaging) and anatomical waste (e.g., placenta).	100			42			71
3.The health facility has at least one functioning hand hygiene station per 10 beds, with soap and water or alcohol-based hand rubs, in all wards.	100			54			77
4.The health facility has energy infrastructure (e.g., solar, generator, grid) that can meet all the electricity demands of the facility and associated infrastructure at all times, with a backup power source.	100						100
6.The health facility has sanitation facilities on premises that are usable, appropriately illuminated at night, accessible to people with limited mobility and separated by gender for staff and patients; they include at least one toilet that meets the needs for menstrual hygiene management, with hand-washing stations and soap and water (at least 1 latrine per 20 users for inpatient settings).	100			82			91
Outcome quality measures							
1.The proportion of women and their families who attended the health facility who were satisfied with the water, sanitation and energy services and would recommend the health facility to friends and family.						67	67
2.The proportion of all health care staff at the health facility who were satisfied with the water, sanitation and energy services and considered that these services contribute positively to providing high-quality care.						42	42

3.The proportion of women and their families who attended the health facility who were satisfied with the power and lighting source and would recommend the health facility to friends and family.						91	91
Average quality statement 8.1							80
Quality statement 8.2 Areas for labour, childbirth and postnatal care are designed, organized, and maintained so that every woman and newborn can be cared for according to their needs in private, to facilitate the continuity of care.							
Input quality measures							
1.The health facility has a dedicated area in the labour and childbirth area for resuscitation of newborns, which is adequately equipped with a table or resuscitative, radiant warmer, light, and appropriate resuscitation equipment and supplies.	100		100	74			91
2.The health facility has a labour ward and an adequate number of birthing rooms or areas for the estimated number of births in the service area.	100	100		100			100
4.The health facility practises and enables rooming-in for all women to allow mothers and babies to remain together 24 h a day.		100					100
5.The health facility has a labour and childbirth area or room with a functional, clean, and accessible bathroom or shower room and toilet for use only by women in labour.	100	100					100
7.The facility has a dedicated recovery room or area for care of women with complications.	100						100
8.The health facility has a dedicated ward for admitting sick and unstable small babies.	100						100
Output/process quality measures							
1.The proportion of all pregnant women who attended the health facility who reported that it has a clean physical environment conductive for childbirth.						42	42
Average for quality statement 8.2							90
Quality statement 8.3Adequate stocks of medicines, supplies and equipment are available for routine care and management of complications							
Input quality measures							
1.The health facility has supplies of antihypertensive agents and magnesium sulphate in sufficient quantities, available at all times, in antenatal, labour, childbirth and postnatal areas for the management of women with pre-eclampsia.	100						100
2.The health facility has uterotonic drugs and supplies for intravenous infusion (syringes, needles, infusion sets, intravenous fluid solutions and blood) available in sufficient quantities at all times in the childbirth and postnatal care areas for the management of women with postpartum haemorrhage.	100		74			87	87
3.The health facility has supplies of antenatal corticosteroids (dexamethasone or betamethasone), antibiotics and magnesium sulphate available in sufficient quantities at all times to manage preterm births.	100		100			51	84

4.The health facility has functioning essential equipment and supplies for the detection of complications (e.g., thermometers, sphygmomanometers, foetal stethoscopes, urine dipsticks, pulse oximeter) in sufficient quantities at all times in the labour and childbirth areas of the maternity unit.	100			89		87	92
5.The health facility has supplies of first- and second-line injectable antibiotics and other essential medicines available at all times for the management of women and newborns with, or at risk for, infections during labour, childbirth, and the early postnatal period.	100						100
6.The health facility has essential laboratory supplies and tests (blood glucose, haemoglobin or packed cell volume, blood group and cross-matching, bilirubin, urine protein, full blood count, blood culture, electrolytes, renal and liver function tests, syphilis, HIV, and malaria rapid diagnostic tests) to support the management of women and newborns.	100			89		87	92
7.The health facility has essential supplies and functioning equipment (including childbirth beds, vacuum, forceps, incubators, weighing machine, sterile gloves) available in sufficient quantities at all times in the labour and childbirth areas.	100			58			79
8.The health facility has supplies and functioning equipment for the emergency care and resuscitation of women (well-stocked resuscitation trolley, suction device, pulse oximeter, airways, laryngoscope, endotracheal tubes, adult bag valve masks, infusion sets, intravenous fluids) available in sufficient quantities all times in areas designated for labour, childbirth, and postnatal care.	100			74			87
9.The health facility has a safe, uninterrupted oxygen source and delivery supplies (nasal prongs, catheters, and masks), including nasal continuous positive airway pressure, available at all times in labour, childbirth and neonatal areas and the operating theatre (when available).	100			84			92
10.The health facility has supplies and functioning equipment for emergency care and resuscitation of newborns (resuscitation table, well-stocked neonatal resuscitation trolley, warmer, suction device, pulse oximeter, laryngoscope) available all times in areas designated for labour, childbirth, and neonatal care.	100			74			87
11.The health facility has an on-site pharmacy and a medicine and supplies stock management system managed by a trained pharmacist or dispenser.	100						100
Output/process quality measures							
1. Availability of essential life-saving medicines (oxytocin, magnesium sulphate, dexamethasone, vitamin K, injectable and oral amoxicillin, benzyl penicillin, gentamicin, ceftriaxone, metronidazole, antimalarial drugs, antiretroviral drugs and vaccines against tuberculosis, hepatitis B, poliomyelitis) in the past three months.	100		100	95	88	42	85
3.The proportion of all women who gave birth in the health facility who purchased gloves and other necessary items.						0	0
Average quality statement 8.3							83

S2 Table. Implemented interventions of WHO/UNICEF maternal and newborn quality improvement standard measures under review by EMEN tools. Green boxes indicate high facility practices of measures included in that quality statement, e.g., score of 80-100%; Yellow boxes indicates moderate practices of quality measures within the quality statement, e.g., 50-79%; and Red boxes indicates the low/poor facility practices of quality measures within the quality statement e.g., 0-49%
