# Supplementary material for: Baseline assessment of the WHO/UNICEF/UNFPA maternal and newborn quality-of-care standards around childbirth: Results from an intermediate hospital, northeast Namibia
Source: Front Pediatr. 2023 Jan 9;10:972815. doi: 10.3389/fped.2022.972815 (PMC9869061; doi:10.3389/fped.2022.972815)
Supplement: Supplementary file 4 [file Datasheet4.pdf]

S4Table. Summary of good interventions and areas of concerns captured in the study

Aspects of care	Good healthcare interventions	Areas of concern
**Infrastructure and supplies**	**Admission:**<br>• There is a separate maternity ward with beds, clientele toilets, adequate supplies, medicine<br>**Labour and delivery:**<br>• There is a separate maternity ward with beds, clientele toilets, adequate supplies, medicines.<br>**Delivery:**<br>• Adequate beds available for all women and babies<br>**Immediate after birth:**<br>• Adequate supplies of stock including cord clamps, weighing scales, thermometers, vitamin K, vaccines.<br>• There is a separate premature unit within maternity ward for low weight babies and premature babies.<br>**Postnatal care:**<br>• Vaccines available.<br>• Beds/mattresses available for women and babies.	**Referral:**<br>• Few items in some ambulances needed to be fixed including stretcher bed, aircon, Haemoglobin bilirubin machine, HGT (Heamoglucose test) machine, and flow meter.
**Enabling policy environment Healthcare improvement**	**Labour/delivery/postnatal**:<br>• Facility has policies on baby friendly, early initiation of breastfeeding, rooming in, Kangaroo Mother Care, Free emergency care, Free delivery care, systematic mortality audits, Systematic "near miss" audits<br>• Infection prevention and control<br>• Suggestion box available	

	• The facility operates 24 hours.	
**Human resources for healthcare improvement**	**Delivery:**<br>• All deliveries were conducted by skilled providers (nurse midwives).<br>• Maternity ward and premature unit have their own staff and duty roster.	**labour/delivery/postnatal:**<br>• Above half of the staff were not trained or refreshed in the past 12 months on management of obstetric emergencies, postnatal care and breastfeeding maternal and newborn complications.
**Clinical healthcare improvement interventions**	**Admission:**<br>• Complete initial examination: blood pressure, temperature, feta heart rate, urine tested, abdominal examination conducted for most of the women.<br>**Immediate after birth:**<br>• Oxytocic administered in most of the women.<br>• Four essential newborn care provided to most of the newborns (baby dried immediately and thoroughly, baby placed in skin-to-skin contact with the mother immediately after birth, delayed cord clamping 1-3 minutes, baby put to breastfeed within 1 hour after birth).<br>• Vitamin K administered to all babies.<br>• Examination of most babies immediate after birth.	**Admission:**<br>Incomplete initial examination: few women checked eyelid/tongue/nails for anaemia<br>**Delivery:**<br>Partograph not filled for most women (only 11%)<br>**Postnatal care:**<br>• Close to forty percent of baby not examined at discharge.<br>• Most women not informed or counselled on breastfeeding, postpartum care and hygiene, family planning.<br>• Most women not educated/informed about maternal and newborn danger signs and when to go to the nearest health facility if any danger sign is present.

	**Postnatal care:**<br>• Immunisation given to all babies.<br>• Above half of the babies examined at discharge.	
**Provider-client interactions**	**Admission:**<br>• Most women were happy with the level of privacy and confidential maintained by providers when talking and/ or examining them.<br>**Labour:**<br>• Most women were happy with the level of privacy and confidential maintained by providers when talking and/ or examining them.	**Admission:**<br>• Providers did not tell most of the women what they found during examinations.<br>**Labour:**<br>• Providers did not allow women a companion<br>**Delivery:**<br>• Providers did not allow women a companion.<br>• Caesarean section procedure not explained to most of the women who underwent surgery/CS.<br>**Postnatal care:**<br>• Most women were not happy with the information they received from providers on breastfeeding, postpartum care and hygiene and family planning.<br>• Providers did not inform most of the mothers on maternal and newborn danger signs.

**Observed healthcare improvement interventions**	**Immediate after birth:**<br>• Oxytocic administered in most of the women.<br>• Four essential newborn care provided to most of the newborns (baby dried immediately and thoroughly, baby placed in skin-to-skin contact with the mother immediately after birth, delayed cord clamping 1-3 minutes, baby put to breastfeed within 1 hour after birth).<br>• Vitamin K administered to all babies.<br>• Examination of most babies.<br>**Postnatal care:**<br>• Immunisation given to all babies.<br>• Above half of the babies examined at discharge.	**Delivery:**<br>• No companion was present for all delivered women<br>• Immediate care after birth<br>**Postnatal care:**<br>• Delivery plan was not communicated to most women.<br>• No women were informed or counselled on breastfeeding, postpartum care and hygiene, family planning.<br>• No mothers were told about maternal and newborn danger signs and when to go to the nearest health facility if any danger sign is present.
**Client perspectives**	Most clients were satisfied with the healthcare services, care provided to their neonates, attitude of the healthcare providers, that they were treated with respect. Most women reported that providers were responsive when asked for support.	Most clientele were not satisfied with the hygiene standard of the health facility, and information given to them on breastfeeding, postpartum care and hygiene and family planning.
