# Supplementary material for: Baseline assessment of the WHO/UNICEF/UNFPA maternal and newborn quality-of-care standards around childbirth: Results from an intermediate hospital, northeast Namibia
Source: Front Pediatr. 2023 Jan 9;10:972815. doi: 10.3389/fped.2022.972815 (PMC9869061; doi:10.3389/fped.2022.972815)
Supplement: Supplementary file 5 [file Datasheet5.pdf]

**S5 Table. Implementation comments on WHO/UNICEF/UNFPA quality improvement standards for maternal and newborn care**	
Quality measures	***Comment***
Output/process 4.2.2 The proportion of all women who gave birth in the health facility who reported that health care staff introduced themselves and showed **good knowledge of the women's history** and the care that had been given to date.	The second part of the indicator can be revised to accommodate some settings where there is a disconnect between providers for antenatal care and those attending to labour and childbirth. In the Namibian context, those in maternity wards rely heavily on the notes written in the antenatal card which is the card the woman brings along to the hospital for delivery. In this case expectation on the "good knowledge of the women's history…" is depended on the notes captured in the card. Antenatal care is done only in primary health care facilities for state clients, and for private clients this may be different.
Output/process 5.2.2 The proportion of women who gave birth in the health facility who were satisfied that the facility met their **religious** and **cultural** needs.	The Namibia health system policy and interventions are such that care is provided regardless of religious and cultural background. In this way care is non-discriminatory and does not favour based on individual faiths or beliefs.
Propose to consider under Standard1. Quality statement 1.1a women are assessed routinely on admission and during labour and childbirth and are given timely, appropriate care.	In low resource setting or in the absence of laboratory facilities, an indicator to check for eyelids/tongue/nails for anaemia may be an option for early identification of anaemia at admission as the facility awaits laboratory confirmation.<br>Our exit interview tool has the capacity to measure an indicator on anaemia during physical examination at admission, and the practice was low. Though we could not match the response to any of the three elements of care (inputs/process/output/outcomes).
